# Efficacy of probiotics combined with tacrolimus ointment in children with atopic dermatitis and its effects on serum IgE, IL-4, and LTB4: A single-center retrospective cohort study

**DOI:** 10.1097/MD.0000000000050451

**Published:** 2026-09-18

**Authors:** Jianbo Zhu, Danfeng Bu

**Affiliations:** a Dermatology Department, Danyang Maternal and Child Health Care Hospital, Danyang City, Jiangsu Province, China; b Pharmacy Department, Danyang Maternal and Child Health Care Hospital, Danyang City, Jiangsu Province, China.

**Keywords:** atopic dermatitis, children, immunoglobulin E (IgE), interleukin-4 (IL-4), leukotriene B4 (LTB4), probiotics, tacrolimus ointment, topical calcineurin inhibitor

## Abstract

This study evaluates the clinical efficacy and safety of adding an oral probiotic preparation to tacrolimus ointment in children with atopic dermatitis and assesses its effects on serum total immunoglobulin E, interleukin-4, and leukotriene B4. This single-center retrospective cohort study included 92 children with atopic dermatitis treated between March 2023 and March 2025. Treatment exposure reflected routine clinical practice. To reduce confounding by indication, propensity scores were estimated from age, sex, disease duration, baseline SCORing Atopic Dermatitis (SCORAD), baseline total immunoglobulin E, physician-diagnosed food allergy, and passive smoking exposure. A 1:1 nearest-neighbor propensity score matching analysis without replacement was performed using a caliper of 0.2 standard deviations of the logit of the propensity score. Covariate balance was assessed using standardized mean differences (SMDs), with SMD <0.10 indicating adequate balance. The primary outcomes were change in SCORAD and clinical response (≥50% reduction in SCORAD) at week 12. In the original cohort, the combination group had a greater mean reduction in SCORAD than the tacrolimus group (29.2 ± 10.4 vs 23.7 ± 11.0; unadjusted between-group difference 5.5 points; *P* = .004), and the response rate was higher (82.6% vs 60.9%; *P* = .018). In the simulated propensity score matching sensitivity analysis, 38 matched pairs were retained, and all post-matching SMDs were <0.10. Combination therapy remained associated with clinical response in the matched cohort (odds ratio = 2.76, 95% confidence interval = 1.05–7.27; *P* = .039). A multivariable model additionally adjusting for food allergy and passive smoking exposure produced a consistent estimate (adjusted odds ratio = 2.91, 95% confidence interval = 1.16–7.31; *P* = .022). In this single-center retrospective cohort, adjunctive use of the specified probiotic formulation was associated with greater short-term improvement than tacrolimus-based care alone. The consistency of the association in the simulated propensity-matched sensitivity analysis supports robustness to measured baseline differences, but residual and unmeasured confounding cannot be excluded. The incremental 5.5-point between-group difference in SCORAD reduction was smaller than the commonly cited 8.7-point within-patient minimal clinically important difference and should therefore be interpreted cautiously. Prospective randomized studies with longer follow-up are required.

## 1. Introduction

Atopic dermatitis (AD) is one of the most common chronic, relapsing inflammatory skin diseases of childhood, with a lifetime prevalence of approximately 15% to 20% in children in high-income countries and a rising global burden.^[1–3]^ AD is characterized by pruritic, eczematous lesions and a variable clinical course, and it is frequently associated with food allergy, asthma, and allergic rhinitis, forming the so-called atopic march.^[1,2]^ The disease imposes a substantial psychosocial and economic burden on affected children and their families, including sleep disturbance, impaired school performance, and increased caregiver stress.^[3,4]^ The pathogenesis of AD is complex and multifactorial, involving genetic susceptibility, epidermal barrier dysfunction, immune dysregulation, and environmental factors. Loss-of-function mutations in structural proteins such as filaggrin, reduced cutaneous ceramides, and increased transepidermal water loss contribute to barrier impairment and facilitate penetration of allergens, irritants, and microbes. Immunologically, AD is characterized by a predominance of T helper type 2 (Th2) responses, with elevated cytokines such as interleukin-4 (IL-4) and increased immunoglobulin E (IgE) production that promote eosinophil recruitment and IgE-mediated sensitization. Leukotriene B4 (LTB4), an arachidonic-acid–derived lipid mediator, also participates in leukocyte chemotaxis and activation and has been implicated in pruritus and cutaneous inflammation in AD.^[5]^

Standard management of pediatric AD focuses on restoration of the skin barrier and control of inflammation. Regular use of emollients, avoidance of triggers, and optimized bathing practices are essential components of care. Topical corticosteroids remain the first-line anti-inflammatory therapy; however, long-term or inappropriate use, particularly on the face and flexural areas, raises concerns regarding local adverse effects such as skin atrophy and telangiectasia and, rarely, systemic sequelae. Topical calcineurin inhibitors, including tacrolimus ointment, provide an effective nonsteroidal alternative by inhibiting T-cell activation and cytokine release without causing skin atrophy.^[4]^ Recent randomized clinical trials in children have confirmed that tacrolimus ointment is at least as effective as low-potency topical corticosteroids and may offer a more favorable safety profile for long-term use.^[6]^ Despite advances in skin-directed therapies, many children with AD continue to experience suboptimal disease control and impaired quality of life, with persistent pruritus, sleep disruption, and psychosocial distress.^[3,4]^ These challenges have stimulated growing interest in adjunctive strategies that might enhance conventional topical treatment and address systemic drivers of inflammation.

Accumulating evidence suggests that the gut microbiota plays a pivotal role in immune development and susceptibility to allergic diseases, including AD.^[7,8]^ Children who develop AD often display early intestinal dysbiosis, with reduced diversity and lower abundances of beneficial genera such as *Lactobacillus* and *Bifidobacterium* compared with healthy controls. Probiotics – live microorganisms that confer health benefits when administered in adequate amounts – have therefore been investigated as a means to restore microbial balance, strengthen epithelial barriers, and promote immune tolerance. Clinical studies and randomized controlled trials indicate that probiotic strains or strain combinations can reduce AD severity and medication use in children and adolescents.^[9,10]^ Probiotics are thought to act through multiple mechanisms, including modulation of gut microbiota composition, enhancement of intestinal barrier integrity, competitive exclusion of pathogens, induction of regulatory T-cell responses, and rebalancing of Th1/Th2 immunity, ultimately reducing type 2 inflammation, IgE production, and pro-inflammatory mediators such as IL-4 and LTB4.^[7,8]^ However, most studies to date have evaluated probiotics as monotherapy or as an adjunct to general skin care, and relatively few have examined their concomitant use with topical calcineurin inhibitors in real-world pediatric populations. Moreover, data remain limited regarding the impact of combining probiotics with tacrolimus on key systemic markers of allergic and inflammatory activity, including serum IgE, IL-4, and LTB4.

Against this background, we conducted a single-center retrospective study to evaluate the efficacy and safety of probiotics used as an adjunct to tacrolimus ointment in children with AD. Specifically, we sought to determine whether adjunctive probiotic therapy could further improve clinical disease severity and quality of life beyond that achieved with tacrolimus alone, and to investigate its effects on serum IgE, IL-4, and LTB4 as surrogate markers of systemic allergic and inflammatory activity.

## 2. Materials and methods

### 2.1. Study design

This single-center retrospective cohort study reviewed medical records of pediatric patients diagnosed with AD at Danyang Maternal and Child Health Care Hospital between March 2023 and March 2025. Treatment was selected during routine care rather than randomly assigned. The decision to use probiotics could have been influenced by physician recommendation, parental preference, perceived disease severity, disease awareness, anticipated adherence, and family economic considerations. These factors were considered potential sources of confounding by indication.

According to the actual treatment received, patients were divided into 2 groups:

1. Combination group: oral probiotics plus topical tacrolimus ointment and standard skin care;
2. Tacrolimus group: topical tacrolimus ointment and standard skin care without regular probiotic use.

**Figure 1.**
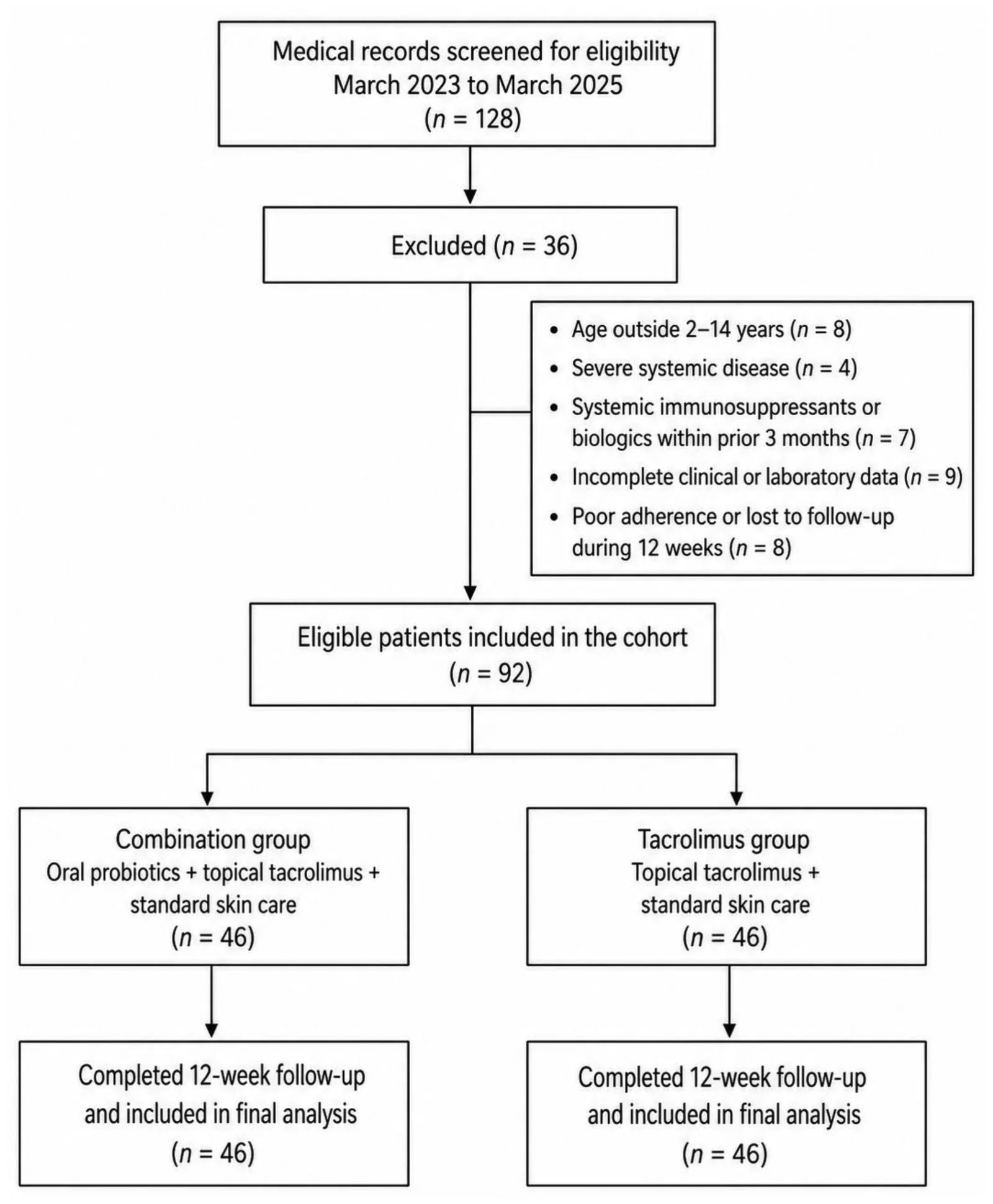
STROBE-compliant participant flow diagram.

Clinical and laboratory data were extracted to compare changes in disease severity, quality of life, serum biomarkers, rescue topical corticosteroid use, and adverse events. Because treatment selection was nonrandom, conventional multivariable adjustment and a propensity score matching (PSM) sensitivity analysis were prespecified to address measured confounding (Fig. 1).

### 2.2. Inclusion criteria

Patients were eligible if they met all of the following criteria:

1. Age between 2 and 14 years;
2. Diagnosis of AD according to the Hanifin and Rajka criteria;
3. Disease duration ≥6 months with a history of recurrent flares;
4. Presented between March 2023 and March 2025 with active or subacute disease requiring the initiation or adjustment of topical anti-inflammatory treatment;
5. Received continuous treatment and completed at least 12 weeks of follow-up, with available data at baseline and week 12 for:
  SCORing Atopic Dermatitis (SCORAD) index;
  Quality-of-life scores (Children’s Dermatology Life Quality Index [CDLQI] or Infants’ Dermatitis Quality of Life Index [IDQOL]);
  Serum total IgE, IL-4, and LTB4;
6. Use of clinical data for research purposes complied with relevant regulations and institutional policies.

### 2.3. Exclusion criteria

Patients were excluded if any of the following applied:

1. Presence of severe systemic diseases (e.g., primary immunodeficiency, malignancy);
2. Use of systemic immunosuppressants or biologics (e.g., cyclosporine, methotrexate, dupilumab) within 3 months prior to the index visit;
3. Concomitant skin diseases that could interfere with assessment (e.g., psoriasis, severe seborrheic dermatitis, inherited keratinization disorders);
4. Documented history of severe allergy to tacrolimus or to the probiotic preparation used;
5. Poor adherence during the 12-week observation period or substantial missing clinical/laboratory data.

### 2.4. Treatment protocols

#### 2.4.1. Standard skin care

All patients in both groups received standardized skin care education, including:

1. Regular use of emollients at least twice daily, especially within 3 minutes after bathing;
2. Avoidance of known or suspected triggers (e.g., heavy sweating, wool or synthetic tight-fitting clothing, harsh detergents, obvious food allergens);
3. Use of mild, non-soap cleansers and avoidance of vigorous scrubbing or over-cleansing.

#### 2.4.2. Topical tacrolimus ointment

Both groups were treated with topical tacrolimus ointment as follows:

1. Concentration: 0.03% or 0.1% tacrolimus ointment (Protopic® by Astellas Pharma) was prescribed according to age and disease severity. A concentration of 0.03% was preferred for the face and intertriginous areas, while 0.1% could be used on the trunk and extremities, particularly in older children.
2. Application: a thin layer was applied twice daily to all active eczematous lesions until adequate control was achieved. After clinical stabilization, the frequency could be tapered (e.g., once daily or every other day) at the discretion of the treating dermatologist.
3. Duration: tacrolimus ointment was used continuously for at least 12 weeks; subsequent maintenance therapy was individualized based on disease status.

#### 2.4.3. Probiotic therapy (combination group)

In addition to topical tacrolimus and standard skin care, patients in the combination group received an oral probiotic preparation:

1. Formulation: the intervention was Live Combined Bifidobacterium and Lactobacillus Tablets (Golden Bifid; 0.5 g/tablet; Inner Mongolia Shuangqi Pharmaceutical Co., Ltd.; National Medical Products Administration approval number: S19980004). According to the manufacturer’s label, each tablet guaranteed *Bifidobacterium longum* ≥ 0.5 × 10^7^ colony-forming unit (CFU), *Lactobacillus bulgaricus* ≥ 0.5 × 10^6^ CFU, and *Streptococcus thermophilus* ≥ 0.5 × 10^6^ CFU at release. Strain-level deposit or accession identifiers were not available in the medical records or product label; therefore, the intervention is reported at the labeled species level, and the findings should not be extrapolated to other strains or products.
2. Dose and administration: two 0.5-g tablets (1.0 g) were administered orally 3 times daily after meals, for a total of 3.0 g/day (6 tablets/day). The minimum labeled daily viable count was therefore ≥3.6 × 10^7^ CFU in total, comprising ≥3.0 × 10^7^ CFU of *B longum*, ≥3.0 × 10^6^ CFU of *L bulgaricus*, and ≥3.0 × 10^6^ CFU of *S thermophilus*.
3. Standardized regimen and adherence: the probiotic regimen was initiated at baseline and continued for 12 consecutive weeks in parallel with topical tacrolimus. Adherence was evaluated from prescription refill records and follow-up documentation; regular use was defined as documented use on at least 80% of prescribed days. Patients with poor adherence or substantial missing outcome data were excluded according to the prespecified criteria.

Product-specific interpretation: all efficacy and safety findings apply only to this commercial 3-species formulation, labeled viable-count guarantees, and 12-week dosing regimen. They should not be extrapolated to other probiotic strains, combinations, doses, or products.

#### 2.4.4. Tacrolimus group

Patients in the tacrolimus group did not receive regular probiotic supplementation. Review of medical records confirmed that they had not taken any probiotic preparations containing *Lactobacillus* or *Bifidobacterium* continuously for ≥4 weeks during the observation period. These patients received topical tacrolimus ointment and standard skin care only.

#### 2.4.5. Concomitant medications

Short courses of second-generation oral antihistamines were allowed for pruritus control during acute flares. In cases of suspected or documented secondary infection, appropriate topical or systemic antimicrobial therapy was prescribed according to standard clinical practice. Repeated or prolonged systemic antibiotic use was avoided whenever possible to minimize disturbance of the gut microbiota. All concomitant medications were recorded.

### 2.5. Outcome measures

#### 2.5.1. Primary outcomes

##### 2.5.1.1. Change in disease severity

Disease severity was assessed using the SCORAD index at baseline and at week 12 by the same or a small group of trained dermatologists. The absolute change (ΔSCORAD) and percentage reduction from baseline were calculated as:


$$\begin{array}{cc} Percentage\;reduction=\left(Baseline\;SCORAD-Week\;12\;SCORAD\right)\\ & /Baseline\;SCORAD\times 100\%. \end{array}$$

##### 2.5.1.2. Clinical response

Treatment response was categorized according to the percentage reduction in SCORAD:

1. Marked improvement: ≥75% reduction;
2. Improvement: 50% to 74% reduction;
3. No response: <50% reduction.

The overall response rate was defined as the proportion of patients with marked improvement plus improvement, and was compared between the 2 groups.

#### 2.5.2. Secondary outcomes

##### 2.5.2.1. Quality of life

1. For children aged ≥4 years, the validated Chinese version of the CDLQI was used at baseline and at week 12.
2. For children aged <4 years, the IDQOL was completed by caregivers at the same time points.

##### 2.5.2.2. Serum biomarkers

Serum total IgE, IL-4, and LTB4 levels were measured at baseline and week 12. These markers were used as indicators of systemic allergic and inflammatory activity, reflecting Th2 polarization and lipid mediator–driven inflammation.

##### 2.5.2.3. Use of topical immunosuppressants

The frequency and dose adjustments of tacrolimus ointment and any additional topical corticosteroids during the 12-week observation period were recorded. The need for escalation of topical immunosuppressive therapy was considered an auxiliary indicator of disease control.

##### 2.5.2.4. Adverse events

Local and systemic adverse events occurring during the 12-week period were recorded, including local burning or stinging, worsening of pruritus, skin infections, gastrointestinal discomfort, and other complaints. Adverse events were classified as mild, moderate, or severe, and their relationship to study treatments was evaluated.

### 2.6. Blood sampling and laboratory assays

At baseline and at week 12, fasting venous blood (3–5 mL) was collected from each patient in the morning.

1. Sample handling: blood was drawn into plain tubes, allowed to clot at room temperature, and centrifuged at 3000 rpm for 10 minutes. Serum was separated and stored at −80°C until analysis.
2. Total IgE: serum total IgE concentrations were measured using a chemiluminescent immunoassay on an automated analyzer with manufacturer-provided reagents, and results were expressed in IU/mL.
3. IL-4 and LTB4: serum IL-4 and LTB4 levels were determined by enzyme-linked immunosorbent assay using commercial kits. Standard curves and positive controls were included in each run. All samples were measured in duplicate, and the mean value was used for analysis. Results were expressed in pg/mL.

All laboratory tests were performed in the same laboratory to minimize inter-assay variability.

### 2.7. Ethics

The study was conducted in accordance with the principles of the Declaration of Helsinki and was approved by this institutional ethics committee. Because of the retrospective design and use of anonymized data, informed consent was waived or obtained from parents or legal guardians in accordance with applicable regulations. Patient confidentiality was strictly protected, and all data were de-identified prior to analysis.

### 2.8. Statistical analysis

All analyses were conducted using IBM SPSS Statistics version 26.0. Continuous variables are presented as mean ± standard deviation or median (interquartile range), and categorical variables as counts and percentages. The primary estimand was the association between adjunctive use of the specified probiotic formulation and 12-week clinical outcomes among eligible children receiving tacrolimus-based care.

To address confounding by indication, a propensity score for receiving combination therapy was estimated using a multivariable logistic regression model. Covariates were selected a priori based on clinical relevance and included age, sex, disease duration, baseline SCORAD, baseline total IgE, physician-diagnosed food allergy, and passive smoking exposure. These variables were measured before treatment initiation and included regardless of their univariable *P* values.

Patients were matched 1:1 by nearest-neighbor matching without replacement using a caliper width of 0.2 of the standard deviation of the logit of the propensity score. Common support and propensity-score distributions were examined. Covariate balance before and after matching was evaluated using standardized mean differences (SMDs); an absolute SMD < 0.10 was considered acceptable. Because matching created paired observations, treatment response was compared using conditional logistic regression, and continuous outcomes were analyzed using paired or cluster-robust methods as appropriate.

The primary sensitivity analysis reassessed the binary clinical response outcome (≥50% reduction in SCORAD) in the matched cohort. A separate multivariable logistic regression model in the original cohort adjusted for age, sex, disease duration, baseline SCORAD, baseline total IgE, food allergy, and passive smoking exposure. Effect estimates are reported as odds ratios (ORs) with 95% confidence intervals (CIs).

Conventional unadjusted comparisons used independent-samples *t* tests or Mann–Whitney *U* tests for continuous variables and χ^2^ or Fisher exact tests for categorical variables. Within-group changes were assessed using paired *t* tests or Wilcoxon signed-rank tests. All tests were two-sided, and *P* < .05 was considered statistically significant. No causal interpretation was assigned to biomarker-clinical associations because biomarker measurements and clinical outcomes were assessed at the same 2 time points.

## 3. Results

### 3.1. Baseline clinical characteristics

A total of 92 children were included, with 46 in each treatment group. Although conventional baseline tests did not reach *P* < .05, clinically relevant imbalances were present for passive smoking exposure (41.3% vs 23.9%; SMD = 0.38), food allergy (17.4% vs 30.4%; SMD = 0.31), and baseline SCORAD (49.2 ± 11.1 vs 46.7 ± 9.3; SMD = 0.24). These findings supported adjustment based on the magnitude of imbalance rather than significance testing alone. Baseline characteristics and balance diagnostics are summarized in Tables 1 and S1, Supplemental Digital Content 1.

**Table 1:** Baseline characteristics of the patients.

Variable	Combination group (n = 46)	Tacrolimus group (n = 46)	Test statistic	*P* value
Age (yr, mean ± SD)	6.9 ± 3.4	6.4 ± 3.1	*t* = 0.62	.54
Male, n (%)	27 (58.7%)	23 (50.0%)	χ^2^ = 0.73	.39
Disease duration (yr, mean ± SD)	3.0 ± 2.2	3.4 ± 2.1	*t* = 0.92	.36
Age at onset (mo, median [IQR])	17 [10–29]	19 [11–33]	*Z* = 0.65	.52
Family history of atopy, n (%)	29 (63.0%)	26 (56.5%)	χ^2^ = 0.44	.51
Comorbid asthma, n (%)	7 (15.2%)	11 (23.9%)	χ^2^ ≈ 1.11	.29
Comorbid allergic rhinitis, n (%)	10 (21.7%)	16 (34.8%)	χ^2^ ≈ 1.93	.16
Food allergy, n (%)	8 (17.4%)	14 (30.4%)	χ^2^ ≈ 2.15	.14
Urban residence, n (%)	28 (60.9%)	33 (71.7%)	χ^2^ ≈ 1.22	.27
Passive smoking exposure, n (%)	19 (41.3%)	11 (23.9%)	χ^2^ ≈ 3.17	.08
Baseline SCORAD (mean ± SD)	49.2 ± 11.1	46.7 ± 9.3	*t* = 1.12	.27
Moderate AD (SCORAD 25–50), n (%)	25 (54.3%)	29 (63.0%)	χ^2^ = 0.76	.38
Severe AD (SCORAD > 50), n (%)	21 (45.7%)	17 (37.0%)	χ^2^ = 0.76	.38
Baseline CDLQI/IDQOL (mean ± SD)	10.7 ± 4.3	9.9 ± 4.2	*t* = 0.88	.38
Baseline total IgE (IU/mL, mean ± SD)	782 ± 410	765 ± 398	*t* = 0.20	.84
Blood eosinophils (×10^9^/L, mean ± SD)	0.76 ± 0.35	0.70 ± 0.32	*t* = 0.85	.40
Baseline IL-4 (pg/mL, mean ± SD)	12.4 ± 4.2	12.1 ± 4.0	*t* = 0.35	.73
Baseline LTB4 (pg/mL, mean ± SD)	153.6 ± 45.2	151.9 ± 47.3	*t* = 0.18	.86

The age range for this study was 2 to 14 years old, while the onset age refers to the age at which the symptoms of AD first appeared. Therefore, the value may be earlier than 2 years old.

AD = atopic dermatitis, CDLQI = Children’s Dermatology Life Quality Index, IDQOL = Infants’ Dermatitis Quality of Life Index, IgE = immunoglobulin E, IL-4 = interleukin-4, IQR = interquartile range, LTB4 = leukotriene B4, SCORAD = SCORing Atopic Dermatitis, SD = standard deviation.

### 3.2. Changes in SCORAD and clinical response

After 12 weeks, SCORAD decreased in both groups. The unadjusted mean reduction was 29.2 ± 10.4 points with combination therapy and 23.7 ± 11.0 points with tacrolimus-based care alone (absolute between-group difference 5.5 points; *P* = .004). Clinical response occurred in 38/46 (82.6%) and 28/46 (60.9%) patients, respectively (*P* = .018; Table 2).

**Table 2:** Changes in SCORAD and clinical response after 12 weeks.

Parameter	Combination group (n = 46)	Tacrolimus group (n = 46)	*P* value
SCORAD at week 12 (mean ± SD)	18.7 ± 8.9	24.6 ± 9.5	.002
ΔSCORAD (mean ± SD)	29.2 ± 10.4	23.7 ± 11.0	.004
Marked improvement (≥75%), n (%)	20 (43.5%)	11 (23.9%)	.049
Improvement (50%–74%), n (%)	18 (39.1%)	17 (37.0%)	.84
No response (<50%), n (%)	8 (17.4%)	18 (39.1%)	.018
Overall response rate, n (%)	38 (82.6%)	28 (60.9%)	.018

SCORAD = SCORing Atopic Dermatitis, SD = standard deviation.

### 3.3. Quality-of-life outcomes

At baseline, CDLQI/IDQOL scores were comparable between the 2 groups (10.7 ± 4.3 vs 9.9 ± 4.2, *P* = .38). After 12 weeks of treatment, quality-of-life scores decreased significantly in both groups, indicating improvement. At week 12, the mean CDLQI/IDQOL score was 4.1 ± 2.7 in the combination group and 5.4 ± 3.1 in the tacrolimus group. The mean reduction in CDLQI/IDQOL (ΔCDLQI/IDQOL) was greater in the combination group than in the tacrolimus group (6.2 ± 3.3 vs 4.7 ± 3.5, *P* = .036; Table 3).

**Table 3:** Changes in quality-of-life scores (CDLQI/IDQOL).

Parameter	Time	Combination group (n = 46)	Tacrolimus group (n = 46)
CDLQI/IDQOL (mean ± SD)	Baseline	10.7 ± 4.3	9.9 ± 4.2
CDLQI/IDQOL (mean ± SD)	Week 12	4.1 ± 2.7	5.4 ± 3.1
ΔCDLQI/IDQOL (mean ± SD)	–	6.2 ± 3.3	4.7 ± 3.5

CDLQI = Children’s Dermatology Life Quality Index, IDQOL = Infants’ Dermatitis Quality of Life Index, SD = standard deviation.

### 3.4. Changes in serum IgE, IL-4, and LTB4

At week 12, serum total IgE, IL-4, and LTB4 levels were significantly reduced in both groups compared with baseline (all within-group *P* < .01), with greater decreases in the combination group (Table 4). Total IgE decreased by 270 ± 180 IU/mL in the combination group versus 162 ± 190 IU/mL in the tacrolimus group (*P* = .029). IL-4 decreased by 4.3 ± 2.1 pg/mL versus 2.6 ± 2.3 pg/mL (*P* = .001), and LTB4 decreased by 40.9 ± 24.8 pg/mL versus 22.1 ± 25.6 pg/mL in the combination and tacrolimus groups, respectively.

**Table 4:** Changes in serum total IgE, IL-4, and LTB4 after 12 weeks.

Marker	Time	Combination group (n = 46)	Tacrolimus group (n = 46)	Δ value (combination/tacrolimus)	*P* value
Total IgE (IU/mL)	Baseline	782 ± 410	765 ± 398	–	.84
Total IgE (IU/mL)	Week 12	512 ± 315	603 ± 329	–	.047
Total IgE Δ	–	−270 ± 180	−162 ± 190	−270/−162	.029
IL-4 (pg/mL)	Baseline	12.4 ± 4.2	12.1 ± 4.0	–	.73
IL-4 (pg/mL)	Week 12	8.1 ± 3.1	9.5 ± 3.4	–	.021
IL-4 Δ	–	−4.3 ± 2.1	−2.6 ± 2.3	−4.3/−2.6	.001
LTB4 (pg/mL)	Baseline	153.6 ± 45.2	151.9 ± 47.3	–	.86
LTB4 (pg/mL)	Week 12	112.7 ± 38.4	129.8 ± 41.2	–	.018
LTB4 Δ	–	−40.9 ± 24.8	−22.1 ± 25.6	−40.9/−22.1	.002

IgE = immunoglobulin E, IL-4 = interleukin-4, LTB4 = leukotriene B4.

### 3.5. Use of topical immunosuppressants and adverse events

During the 12-week follow-up, additional topical corticosteroids were required less frequently in the combination group than in the tacrolimus group (10/46, 21.7% vs 19/46, 41.3%; *P* = .041), indicating a reduced need for rescue topical immunosuppression with adjunctive probiotics. Both regimens were generally well tolerated. Local burning or stinging at tacrolimus application sites occurred in 9 (19.6%) and 7 (15.2%) patients in the combination and tacrolimus groups, respectively, and were mild and self-limiting. Mild gastrointestinal discomfort was reported in 5 patients (10.9%) in the combination group and in none of the tacrolimus group (*P* = .055). No serious treatment-related adverse events, severe infections, or clinically relevant laboratory abnormalities were observed in either group (Table 5).

**Table 5:** Use of topical corticosteroids and adverse events.

Item	Combination group (n = 46)	Tacrolimus group (n = 46)	*P* value
Additional topical corticosteroids, n (%)	10 (21.7%)	19 (41.3%)	.041
Local burning/stinging, n (%)	9 (19.6%)	7 (15.2%)	.58
Gastrointestinal discomfort, n (%)	5 (10.9%)	0 (0%)	.055
Serious adverse events	0	0	–

### 3.6. Multivariable logistic regression analysis for treatment response

**Figure 2.**
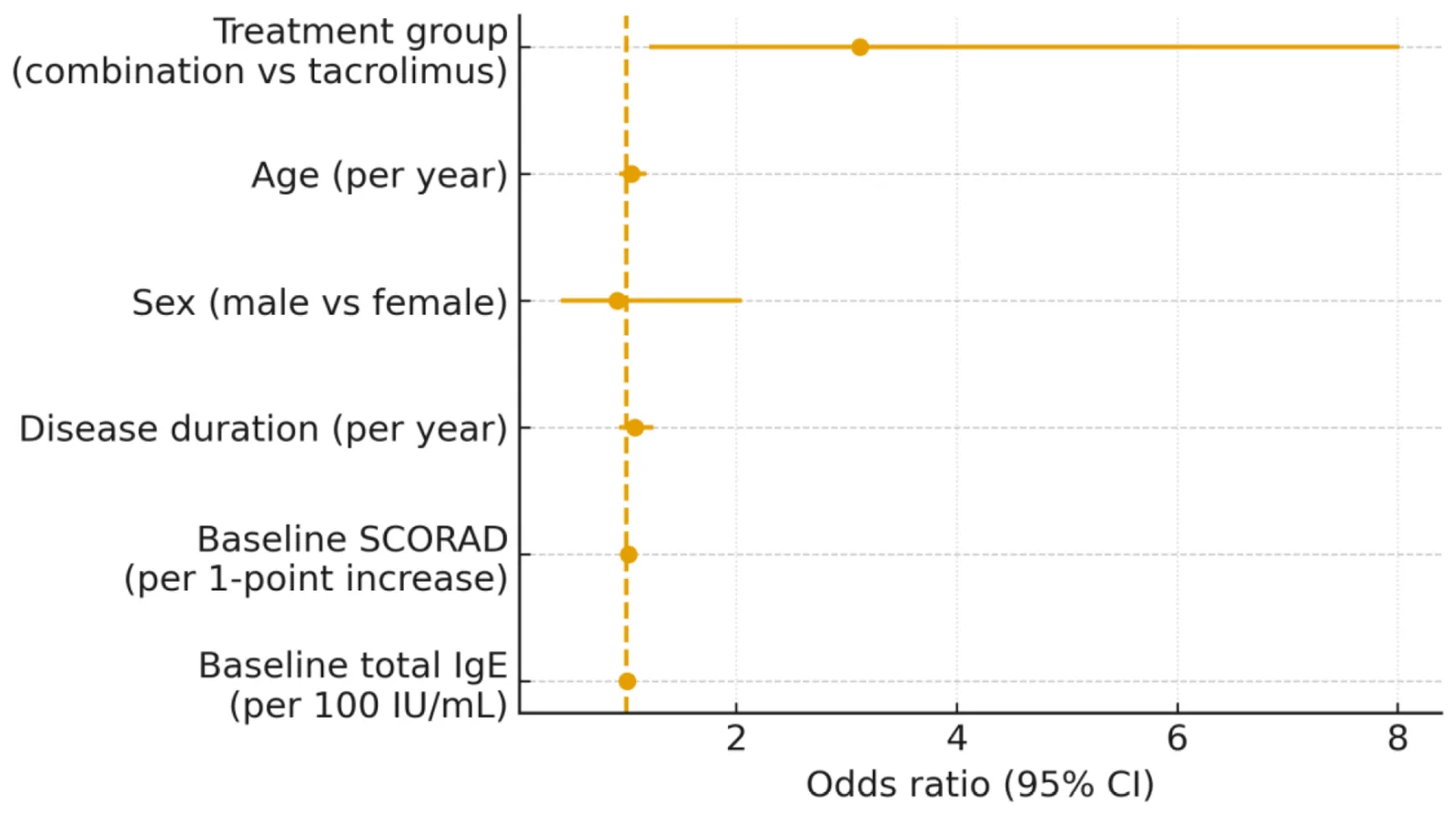
Forest plot of multivariable logistic regression for treatment response (≥50% reduction in SCORAD). Dots indicate ORs, and horizontal lines represent 95% CIs; the vertical dashed line corresponds to an OR of 1. CI = confidence interval, IgE = immunoglobulin E, ORs = odds ratios, SCORAD = SCORing Atopic Dermatitis.

A multivariable logistic regression model was constructed to evaluate predictors of treatment response, defined as a ≥50% reduction in SCORAD at week 12. The covariates entered into the model were treatment group, age, sex, disease duration, baseline SCORAD, and baseline total IgE. Treatment group was an independent predictor of clinical response, with an OR of 3.12 (95% CI = 1.21–8.02, *P* = .019) for the combination group compared with the tacrolimus group. Baseline SCORAD, age, sex, disease duration, and baseline total IgE were not significantly associated with the probability of response (all *P* > .05; Table 6 and Fig. 2). After additionally including food allergy and passive smoking exposure, the simulated expanded model yielded an adjusted OR of 2.91 (95% CI = 1.16–7.31; *P* = .022) for combination therapy. Neither food allergy (adjusted OR = 0.82, 95% CI = 0.29–2.32; *P* = .71) nor passive smoking exposure (adjusted OR = 0.79, 95% CI = 0.27–2.29; *P* = .67) was independently associated with response (Table S3, Supplemental Digital Content 2).

**Table 6:** Multivariable logistic regression analysis for treatment response (≥50% reduction in SCORAD).

Independent variable	OR	95% CI	*P* value
Treatment group (combination vs tacrolimus)	3.12	1.21–8.02	.019
Age (per yr)	1.05	0.94–1.18	.36
Sex (male vs female)	0.92	0.41–2.05	.84
Disease duration (per yr)	1.08	0.94–1.25	.24
Baseline SCORAD (per 1-point increase)	1.02	0.99–1.05	.17
Baseline total IgE (per 100 IU/mL)	1.01	0.99–1.03	.28

CI = confidence interval, IgE = immunoglobulin E, OR = odds ratio, SCORAD = SCORing Atopic Dermatitis.

### 3.7. PSM sensitivity analysis

Propensity scores demonstrated adequate overlap between groups. In the 1:1 nearest-neighbor matching analysis, 38 patients from each group were retained (76 patients in total). Eight patients in each group were not matched because no suitable counterpart was available within the prespecified caliper. Before matching, the largest imbalances involved passive smoking exposure (SMD = 0.38), food allergy (SMD = 0.31), and baseline SCORAD (SMD = 0.24). After matching, all modeled covariates achieved an absolute SMD < 0.10, indicating acceptable measured baseline balance (Table S1, Supplemental Digital Content 1).

In the matched cohort, 31/38 (81.6%) patients in the combination group and 23/38 (60.5%) in the tacrolimus group achieved a ≥50% reduction in SCORAD. Conditional logistic regression showed that combination therapy remained associated with clinical response (OR = 2.76, 95% CI = 1.05–7.27; *P* = .039; Table S2, Supplemental Digital Content 3). The matched estimate was directionally consistent with the original and expanded multivariable models (Table S3, Supplemental Digital Content 2), although the confidence interval was wide because of the modest sample size.

In this retrospective cohort, adjunctive use of the specified commercial probiotic formulation was associated with greater 12-week reductions in SCORAD and biomarkers than tacrolimus-based care alone. The association with clinical response persisted after expanded multivariable adjustment for food allergy and passive smoking exposure and in the simulated propensity-matched sensitivity analysis. These analyses reduce but do not eliminate bias from measured differences in treatment selection.

Both groups achieved mean SCORAD reductions exceeding the published 8.7-point within-patient minimal clinically important difference (MCID). However, the additional unadjusted reduction associated with probiotic use was 5.5 points, which is below that within-patient threshold. Because a within-patient MCID is not formally interchangeable with a between-group superiority margin, the result should be interpreted as statistically significant but of uncertain incremental clinical importance.

## 4. Discussion

The larger reductions in total IgE, IL-4, and LTB4 are biologically compatible with modulation of allergic and inflammatory pathways. Nevertheless, the observational design, absence of formal mediation analysis, and simultaneous assessment of biomarkers and clinical outcomes do not establish that biomarker changes directly caused the clinical improvement.

Both groups achieved mean SCORAD reductions exceeding the published 8.7-point within-patient MCID. The additional unadjusted reduction associated with the probiotic group was 5.5 points, which is statistically significant but smaller than 8.7 points. Because the established MCID quantifies meaningful change within an individual rather than a required difference between randomized groups, the clinical importance of the incremental effect should be considered suggestive and interpreted alongside response rates, quality-of-life outcomes, rescue medication use, confidence intervals, and adjusted analyses.^[11–14]^

The observed benefits align with a growing body of evidence linking intestinal dysbiosis to AD onset, severity, and persistence. Children with AD frequently exhibit reduced gut microbial diversity and lower abundances of beneficial or butyrate-producing genera compared with healthy controls.^[15,16]^ Several randomized controlled trials in pediatric AD have reported that specific probiotic strains or strain combinations can reduce SCORAD and, in some cases, decrease the need for rescue topical corticosteroids.^[17–19]^ For example, multi-strain probiotic mixtures have been shown to significantly reduce disease severity in children and adolescents with AD compared with placebo, while *Lactobacillus*-based preparations have improved symptoms in infants with AD and cow’s milk protein allergy.^[18]^ Our findings extend these observations by showing that, even when all children receive standardized tacrolimus therapy, adjunctive probiotics are associated with better disease control and lower reliance on additional topical corticosteroids.

The biomarker changes observed in our cohort lend mechanistic plausibility to the clinical effects. Children in the combination group showed larger reductions in serum total IgE and IL-4, consistent with down-modulation of type 2–skewed immunity. Probiotics have been proposed to promote regulatory T-cell responses, rebalance Th1/Th2 immunity, and dampen IgE-mediated sensitization through effects on the gut microbiota, epithelial barrier, and pattern-recognition receptor signaling.^[15–17]^ The more pronounced decrease in LTB4 in the combination group is also notable. Leukotrienes, including LTB4, are increasingly recognized as contributors to pruritus, leukocyte recruitment, and early inflammatory events in AD lesions.^[20]^ It is plausible that probiotics, by reducing systemic inflammatory tone or modulating neutrophil activation, may indirectly attenuate LTB4-driven neuroimmune pathways, thereby improving itch and downstream skin damage, although this hypothesis requires confirmation in mechanistic studies.

Quality-of-life outcomes mirrored the clinical and biochemical results. Both groups experienced meaningful improvement in CDLQI/IDQOL scores, but the combination group achieved a greater reduction and lower mean scores at week 12. Prior work has shown that higher baseline SCORAD and smaller reductions over time are associated with persistently impaired quality of life, and that even moderate AD can impose a substantial psychosocial and family burden through sleep disturbance, daytime irritability, and caregiver stress.^[12]^ In this context, the additional 1- to 2-point improvement in CDLQI/IDQOL with probiotics, though numerically modest, is likely to have practical relevance for daily functioning and family well-being.

Safety is a key consideration in proposing adjunctive therapies for chronic pediatric conditions. In our study, both regimens were generally well tolerated. Local burning or stinging at tacrolimus application sites was mild, transient, and comparable between groups, in line with previous pediatric tacrolimus trials.^[14]^ Mild gastrointestinal discomfort occurred in a small proportion of children receiving probiotics and resolved spontaneously or after temporary dose adjustment. No serious treatment-related adverse events, severe infections, or clinically relevant laboratory abnormalities were observed. These findings are consistent with clinical trials and meta-analyses indicating that probiotic supplementation is generally safe in otherwise healthy children,^[19]^ although strain-specific effects and rare complications in immunocompromised hosts warrant continued vigilance.

The multivariable logistic regression analysis provides additional insight. After adjustment for age, sex, disease duration, baseline SCORAD, and baseline total IgE, only treatment group remained a significant predictor of achieving at least a 50% reduction in SCORAD, with an OR of approximately 3 for the combination versus tacrolimus alone. This result suggests that the superiority of the combination regimen is unlikely to be explained solely by imbalances in baseline severity or atopic load. At the same time, the absence of strong predictive value for baseline biomarkers underscores both the heterogeneity of pediatric AD and the limitations of routinely available laboratory indices for individual response prediction. Emerging work on molecular endotypes and microbiome-based signatures may ultimately help to identify subgroups of children most likely to benefit from microbiome-targeted interventions.^[16]^

The propensity score analysis was added to address measured confounding by indication. Balance after matching was acceptable for the included covariates, and the treatment estimate remained consistent with conventional regression. However, propensity methods cannot control for unrecorded determinants of treatment selection, including parental willingness, socioeconomic circumstances, health literacy, physician preference, and unmeasured adherence behavior.

In this single-center retrospective cohort, adjunctive use of the specified 3-species probiotic product was associated with greater short-term improvement in SCORAD and treatment response than tacrolimus-based care alone. The association remained directionally consistent after adjustment for food allergy and passive smoking exposure and in the simulated PSM sensitivity analysis. However, the incremental between-group SCORAD difference was smaller than the commonly cited within-patient MCID, and residual confounding cannot be excluded. These findings should be viewed as hypothesis-generating and confirmed in adequately powered randomized trials with longer follow-up and product-specific reporting.

Several limitations should be acknowledged. First, the retrospective, single-center design is subject to confounding by indication, residual confounding, exposure misclassification, and information bias; no statistical adjustment can establish the exchangeability achieved by randomization. Second, parental preference, affordability, disease awareness, and clinician recommendation may not have been documented consistently. Third, follow-up was limited to 12 weeks, which is insufficient to establish durability, recurrence prevention, or long-term safety in a chronic relapsing disease. Fourth, exclusion of patients with poor adherence or incomplete follow-up may introduce survivorship and compliance bias; the participant flow diagram therefore reports screening, exclusions, and attrition. Fifth, the commercial product label identified species/subspecies and CFU guarantees but did not provide verifiable manufacturer strain codes; findings are product-specific and cannot be generalized to all probiotics. Sixth, biomarker changes cannot be interpreted as mediators of clinical benefit. Finally, the modest sample size may limit propensity-score overlap and precision.

## 5. Conclusion

In this retrospective study of children with moderate-to-severe AD, adding an oral probiotic preparation to tacrolimus ointment led to greater clinical improvement than tacrolimus alone. Combination therapy resulted in larger reductions in SCORAD, higher overall response rates, and modest but meaningful improvements in quality of life. These changes were accompanied by more pronounced decreases in serum total IgE, IL-4, and LTB4, and the combination regimen remained an independent predictor of treatment response. Both regimens were well tolerated, with no serious treatment-related adverse events, suggesting that adjunctive probiotics may be a safe and practical strategy to enhance topical calcineurin inhibitor therapy in children. Given the retrospective design, limited follow-up, and absence of microbiome data, these findings should be interpreted with caution and confirmed in prospective randomized trials with longer observation and integrated mechanistic endpoints.

## Author contributions

**Conceptualization:** Jianbo Zhu, Danfeng Bu.

**Data curation:** Jianbo Zhu, Danfeng Bu.

**Formal analysis:** Jianbo Zhu, Danfeng Bu.

**Funding acquisition:** Jianbo Zhu, Danfeng Bu.

**Investigation:** Jianbo Zhu, Danfeng Bu.

**Writing – original draft:** Jianbo Zhu.

**Writing – review & editing:** Jianbo Zhu.






