# Supplementary material for: Efficacy of probiotics combined with tacrolimus ointment in children with atopic dermatitis and its effects on serum IgE, IL-4, and LTB4: A single-center retrospective cohort study
Source: Medicine (Baltimore). 2026 Sep 18;105(38):e50451. doi: 10.1097/MD.0000000000050451 (PMC13593240; doi:10.1097/MD.0000000000050451)
Supplement: Supplementary file 1 [file medi-105-e50451-s001.docx]

**Supplementary Table S1. Covariate balance before and after propensity score matching**

| **Covariate** | **Before matching: combination group (n = 46)** | **Before matching: tacrolimus group (n = 46)** | **SMD before matching** | **After matching: combination group (n = 38)** | **After matching: tacrolimus group (n = 38)** | **SMD after matching** |
| --- | --- | --- | --- | --- | --- | --- |
| Age, years | 6.9 ± 3.4 | 6.4 ± 3.1 | 0.15 | 6.8 ± 3.2 | 6.6 ± 3.3 | 0.06 |
| Male sex, n (%) | 27 (58.7) | 23 (50.0) | 0.18 | 21 (55.3) | 20 (52.6) | 0.05 |
| Disease duration, years | 3.0 ± 2.2 | 3.4 ± 2.1 | 0.19 | 3.1 ± 2.1 | 3.3 ± 2.2 | 0.09 |
| Baseline SCORAD | 49.2 ± 11.1 | 46.7 ± 9.3 | 0.24 | 48.4 ± 10.5 | 47.6 ± 9.5 | 0.08 |
| Baseline total IgE, IU/mL | 782 ± 410 | 765 ± 398 | 0.04 | 770 ± 390 | 750 ± 410 | 0.05 |
| Food allergy, n (%) | 8 (17.4) | 14 (30.4) | 0.31 | 8 (21.1) | 9 (23.7) | 0.06 |
| Passive smoking exposure, n (%) | 19 (41.3) | 11 (23.9) | 0.38 | 13 (34.2) | 12 (31.6) | 0.06 |

**Note:** Continuous variables are presented as mean ± standard deviation and categorical variables as number (percentage). Absolute SMDs were used to assess covariate balance; values <0.10 were considered acceptable. Matching used 1:1 nearest-neighbor matching without replacement and a caliper of 0.2 of the standard deviation of the logit of the propensity score.
