# Supplementary material for: Efficacy of probiotics combined with tacrolimus ointment in children with atopic dermatitis and its effects on serum IgE, IL-4, and LTB4: A single-center retrospective cohort study
Source: Medicine (Baltimore). 2026 Sep 18;105(38):e50451. doi: 10.1097/MD.0000000000050451 (PMC13593240; doi:10.1097/MD.0000000000050451)
Supplement: Supplementary file 2 [file medi-105-e50451-s002.docx]

**Supplementary Table S3. Sensitivity analyses for clinical response**

| **Analysis** | **OR** | **95% CI** | **P value** |
| --- | --- | --- | --- |
| Original multivariable model | 3.12 | 1.21–8.02 | 0.019 |
| Expanded model including food allergy and passive smoking | 2.91 | 1.16–7.31 | 0.022 |
| Propensity score matched model | 2.76 | 1.05–7.27 | 0.039 |

Abbreviations: OR, odds ratio; CI, confidence interval.
