# Supplementary material for: Efficacy of probiotics combined with tacrolimus ointment in children with atopic dermatitis and its effects on serum IgE, IL-4, and LTB4: A single-center retrospective cohort study
Source: Medicine (Baltimore). 2026 Sep 18;105(38):e50451. doi: 10.1097/MD.0000000000050451 (PMC13593240; doi:10.1097/MD.0000000000050451)
Supplement: Supplementary file 3 [file medi-105-e50451-s003.docx]

**Supplementary Table S2. Treatment response after propensity score matching**

| **Analysis group** | **Matched n** | **Clinical response / effect estimate** |
| --- | --- | --- |
| Combination group | 38 | 31 (81.6%) |
| Tacrolimus group | 38 | 23 (60.5%) |
| Conditional logistic regression | — | OR 2.76 (95% CI 1.05–7.27), P = 0.039 |
